# Mitochondrial dysfunction and increased glycolysis in prodromal and early Parkinson's blood cells

**DOI:** 10.1002/mds.104

**Published:** 2018-10-07

**Authors:** Amy M. Smith, Constanze Depp, Brent J. Ryan, Geoffrey I. Johnston, Javier Alegre‐Abarrategui, Samuel Evetts, Michal Rolinski, Fahd Baig, Claudio Ruffmann, Anna Katharina Simon, Michele T. M. Hu, Richard Wade‐Martins

**Affiliations:** ^1^ Oxford Parkinson's Disease Centre University of Oxford Oxford UK; ^2^ Department of Physiology, Anatomy and Genetics University of Oxford Oxford UK; ^3^ Global Exploratory Development, New Medicines, UCB Pharma Slough UK; ^4^ Nuffield Department of Clinical Neurosciences University of Oxford Oxford UK; ^5^ Translational Immunology Laboratory, NIHR BRC University of Oxford Oxford UK; ^6^ Kennedy Institute of Rheumatology University of Oxford Oxford UK

**Keywords:** Parkinson's disease, peripheral blood mononuclear cells, mitochondrial dysfunction, glycolysis, REM‐sleep behavior disorder

## Abstract

**Background:** Although primarily a neurodegenerative process, there is increasing awareness of peripheral disease mechanisms in Parkinson's disease. To investigate disease processes in accessible patient cells, we studied peripheral blood mononuclear cells in recently diagnosed PD patients and rapid eye movement‐sleep behavior disorder patients who have a greatly increased risk of developing PD. We hypothesized that peripheral blood mononuclear cells may recapitulate cellular pathology found in the PD brain and investigated these cells for mitochondrial dysfunction and oxidative stress.

**Methods:** Peripheral blood mononuclear cells were isolated and studied from PD patients, rapid eye movement‐sleep behavior disorder patients and age‐ and sex‐matched control individuals from the well‐characterized Oxford Discovery cohort. All participants underwent thorough clinical assessment.

**Results:** Initial characterization showed that PD patients had elevated levels of CD14 + monocytes and monocytes expressing C‐C motif chemokine receptor 2. Mitochondrial dysfunction and oxidative stress were increased in PD patient peripheral blood mononuclear cells, with elevated levels of mitochondrial reactive oxygen species specifically in patient monocytes. This was combined with reduced levels of the antioxidant superoxide dismutase in blood cells from PD patients and, importantly, also in rapid eye movement‐sleep behavior disorder patients. This mitochondrial dysfunction was associated with a concomitant increase in glycolysis in both PD and rapid eye movement‐sleep behavior disorder patient blood cells independent of glucose uptake or monocyte activation.

**Conclusions:** This work demonstrates functional bioenergetic deficits in PD and rapid eye movement‐sleep behavior disorder patient blood cells during the early stages of human disease. © 2018 The Authors. Movement Disorders published by Wiley Periodicals, Inc. on behalf of International Parkinson and Movement Disorder Society.

Most cases of Parkinson's disease (PD) are idiopathic, and there is as yet no diagnostic or predictive molecular marker of disease. It has been estimated that at the point of PD diagnosis, there is already significant dopaminergic neuron loss and associated pathology in the substantia nigra, making it difficult to implement disease‐modifying treatments.^1^ There is now substantial evidence that one such group of people who may benefit from disease‐modifying treatments are patients with idiopathic rapid eye movement (REM)‐sleep behavior disorder (RBD), up to 80% of whom will progress to PD or a related synucleinopathy, forming a highly enriched PD at‐risk group.^2^, ^3^, ^4^

As a source of patient tissue in which to study early disease processes, blood samples are easily accessible and cost‐efficient and can be obtained with low risk to the patient. Peripheral blood cells perform many of the fundamental cellular processes that are perturbed in PD, and we hypothesized that they may recapitulate the cellular pathology observed in the PD brain. Several lines of evidence indicate strong immune involvement in PD pathogenesis, including neuroinflammation in PD postmortem brains shown by immunochemistry and PET imaging.^5^, ^6^, ^7^ Several of the mutations that give rise to PD are found in genes that are highly expressed by immune cells including the relatively common genetic mutations in *LRRK2* and *GBA* and the meta‐GWAS hit *HLA‐DQB1*.^8^, ^9^, ^10^ It is less clear, however, whether PD‐related neuronal pathology is reflected or can be detected in peripheral blood cells and whether detection will be sensitive enough to provide a practical indication of early disease processes and progression.

The current article describes a functional study in peripheral blood mononuclear cells (PBMCs) from PD patients, RBD patients, and control individuals from the well‐characterized longitudinal Oxford Discovery cohort.^11^ Focusing on a key pathological mechanism that has been shown to play a role in dopaminergic neuron death, we tested the hypothesis that mitochondrial and bioenergetic changes would be present in PD patient peripheral blood cells. Mitochondrial dysfunction and oxidative stress have been shown to be major pathological mechanisms in PD models ranging from neurotoxin and genetic rodent models to iPSC neurons.^12^, ^13^ Indeed, analysis of Parkinson's patient and aged human postmortem brain tissue has revealed the presence of oxidative species, decreased levels of antioxidants, and elevated markers of oxidative damage and mitochondrial DNA mutations.^12^, ^14^, ^15^ We assessed mitochondrial health using flow cytometry for high‐throughput analysis of large numbers of cells and an Extracellular Flux Analyzer to measure oxygen consumption and glycolysis rates.^16^ In this study we demonstrate functional bioenergetic changes in peripheral cells at the earliest identifiable stages of disease.

## Methods

### Participant Information

This work was carried out in agreement with the Declaration of Helsinki with the approval of the local National Health Service ethics committee and University of Oxford Research Ethics Committee and with written informed consent from every participant. Participants were recruited through the Oxford Parkinson Disease Center Discovery Cohort.^11^, ^17^, ^18^ PD patients were recently diagnosed according to the UK Parkinson's Disease Society Brain Bank criteria. RBD patients were recruited from sleep clinics at John Radcliffe Hospital in Oxford and Papworth Hospital in Cambridge. Idiopathic RBD diagnosis was made as previously described,^17^ according to standard criteria of the International Classification of Sleep Disorders II following overnight polysomnograph. RBD patients who already had dementia, mild cognitive impairment, or parkinsonism at baseline were excluded. Age‐ and sex‐matched control participants were recruited from the same geographical areas and were frequently spouses of patients. Blood samples were collected from individuals who came into the clinic between March 2014 and December 2016 (Table S1). Samples for all experiments were matched across disease groups with no significant differences in age, sex, or blood sample processing time. An outline of the principal experiments is provided in Figure S1, where we indicate the number of donor samples used for each experiment. Initial flow‐cytometric experiments were performed with large numbers of donor samples (n = 50); however, because of reduced availability of samples, subsequent experiments were performed with smaller sample sizes. Patient samples used in these subsequent analyses were selected based on age‐ and sex‐matching criteria for the control samples available and were not selected based on pathological or biochemical criteria.

### PBMC Isolation

Fifteen milliliters of blood from each participant was collected in K_2_‐ethylenediaminetetraacetic acid‐coated vacutainer tubes (BD Biosciences, San Jose, CA; 367839). PBMCs were isolated from whole blood using a standard Ficoll‐Paque Plus gradient solution and centrifugation procedure. Briefly, 15 mL of blood mixed with 10 mL of phosphate‐buffered saline (PBS) pH 7.4 (Gibco, Waltham, MA; 10010023) was slowly pipetted on top of 15 mL of Ficoll‐Paque Plus (GE Healthcare, Amersham, UK; 17‐1440‐03) gradient solution in a 50‐mL Leucosep tube (Greiner Bio‐One, Stonehouse, UK; GREI227290UK) and centrifuged for 10 minutes at 1000*g* with minimal acceleration and deceleration. The “buffy‐coat” layer of PBMCs was aspirated into a new 50‐mL Falcon tube and washed 3 times with 40 mL of PBS, centrifuging at 300*g* for 10 minutes with normal acceleration/deceleration settings. Cells were cryopreserved in 0.5 mL of PBS and 0.5 mL of freeze mix (fetal bovine serum containing 20% dimethylsulfoxide).

### Flow Cytometry

On the day prior to flow‐cytometric analysis, cells were plated in Roswell Park Memorial Institute media (Gibco) containing 10% fetal calf serum (FCS) and 1% l‐glutamine into round bottom 96‐well plates (CLS3799; Sigma, Haverhill, UK). For surface antibody staining, cells were blocked for 10 minutes at 4**°**C with 100 μL of PBS containing 5% FCS and 1% bovine serum albumin. Live/Dead Fixable Near‐IR Dead Cell Stain (L10119; Molecular Probes, Waltham, MA) was used at 1:2000 (15 minutes at 4**°**C). Cells were incubated with surface marker antibodies (Table S2) for 20 minutes at 4**°**C. Data were acquired using a Becton Dickinson LSR II flow cytometer and analyzed using FlowJo vX.0.7. Populations of interest were gated using a combination of unstained, fluorescence‐minus‐one and internal negative population controls as appropriate (Fig. S2).

### MitoSox and MitoTracker

Cells were incubated with 5 μM MitoSox Red (Invitrogen, Waltham, MA) and 100 nM MitoTracker Deep Red (Invitrogen) for 30 minutes at 37°C. PBS was added to compensation controls and unstained sample. Cells were labeled with Live/Dead dye and analyzed by flow cytometry as described above. Rotenone‐treated cells (50 μM, 1.5 hours) were used as an internal control for technical variability between experiments.

### Tetramethylrhodamine Methyl Ester

Two wells were plated per sample, and 1 well was treated with 50 μM carbonyl cyanide *m*‐chlorophenyl hydrazone (CCCP) mitochondrial uncoupler for 1 hour. All cells were incubated with 100 nM tetramethylrhodamine methyl ester (TMRM; Invitrogen, Waltham, MA) for 30 minutes at 37°C. Mitochondrial membrane potential was calculated as basal minus CCCP‐treated.

### Glucose Uptake and GLUT1 Surface Expression

PBMCs were incubated with fluorescent d‐glucose analogue 2‐NBDG (Molecular Probes, Waltham, MA) at 25 µg/mL for 30 minutes. GLUT1 was assessed using GLUT1.RBD (Metafora Biosystems, Evry, France) according to the manufacturer's instructions at 1:100. Binding of the RBD was visualized using R‐phycoerythrin (PE) rat antimouse IgG1 (550083; BD Pharmingen, San Jose, CA).

### Antioxidant Enzyme Activity Assays

Total SOD activity was measured using a Superoxide Dismutase Assay Kit (706002; Cayman Chemical Company, Ann Arbor, MI). Catalase activity was measured using a Catalase Assay Kit (707002; Cayman Chemical Company).

### Reverse Transcription and Quantitative Real‐Time Polymerase Chain Reaction

Quantitative real‐time polymerase chain reaction (qPCR) was carried out using an RNeasy Micro Kit (Qiagen, Manchester, UK), SuperScript III Reverse Transcriptase (Invitrogen), and Fast SYBR Green (Invitrogen) and analyzed using an Applied Biosystems StepOnePlus Real‐Time PCR System. Table S3 lists the primers used. Gene expression was quantified from samples run in triplicate using the 2^‐(ΔΔCT)^ method,^19^ normalized to the mean of 3 endogenous control genes — β‐actin, GAPDH, and β2‐microglobulin — and then normalized to the mean of the healthy control samples.

### Seahorse Extracellular Flux Analyzer

Oxygen consumption rate (OCR) and extracellular acidification rate (ECAR) were measured using a Seahorse XF^e^96 Extracellular Flux Analyzer (Seahorse Biosciences, Billerica, MA) according to the manufacturer's instructions. Experimental setup for PBMCs was based on that in Jones et al,^16^ with minor adjustments. Cells were sequentially treated with 0.5 µM oligomycin, 1 µM FCCP, 0.5 µM rotenone/antimycin A, and 50 mM 2‐deoxyglucose. Data were normalized to DAPI cell counts and then to the mean of the healthy control samples. Analysis was performed using Wave software.

### Fluidigm Gene Expression Analysis

PBMCs were sorted using a BD FACSAria III based on CD14 surface marker expression (CD14‐PE; Miltenyi Biotec, Bisley, UK); monocytes were gated for high CD14 expression, and lymphocytes were selected based on lack of CD14 expression. Duplicate wells of 100 cells were sorted for both monocytes and lymphocytes for each sample. Reverse transcription, specific target amplification, and 48.48 Dynamic Arrays (Fluidigm, Cambridge, UK) were performed as previously described.^20^ A no‐RT and no template control was included in each experiment. Gene expression was quantified using the 2^‐(ΔΔCT)^ method,^19^ normalized to the mean of 2 endogenous control genes, β‐actin and β2‐microglobulin, and then normalized to the internal control sample that was run in each of 3 experiments.

### Statistical Analysis

Graphing and statistical analysis were performed using GraphPad Prism 6. Data are presented with each point representing a participant sample and the number of samples used in each experiment is indicated in the figure legends. Outliers were identified by the ROUT test (Q = 1%) and removed from analysis. Normality was assessed using the D'Agostino & Pearson omnibus normality test, and, according to this outcome, significance was determined by the Mann‐Whitney test or 1‐way analysis of variance (ANOVA). A *P* < 0.05 was considered significant, except for the Fluidigm gene expression analysis. Here, Bonferroni correction was used to adjust the significance thresholds for multiple comparisons (α = 0.001 for monocytes; α = 0.002 for lymphocytes). This method identified the same significant results obtained using the Benjamini‐Hochberg procedure with a false discovery rate of 0.1%. Experiments were performed using the largest sample size possible (determined by availability of patient samples) while maintaining age‐ and sex‐matched groups. Numbers are given in the figure legends.

## Results

### Parkinson's Patients Have Increased Frequency of CD14 + and CCR2 + Monocytes

**Figure 1:**
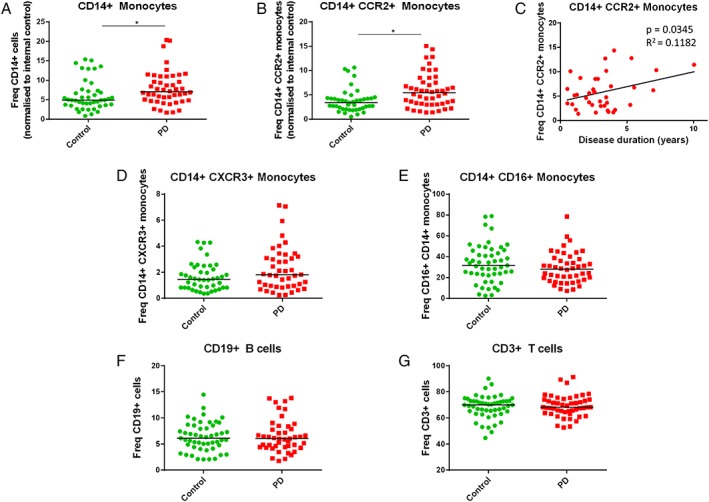
PD patients have greater relative frequencies of monocytes (CD14+) and CCR2 + monocytes compared with controls. Flow cytometry was used to assess the frequencies of different blood cell subtypes in PBMC samples from PD patients and matched controls. (A) CD14 + monocyte population of PBMCs is larger in PD samples compared with controls. An increase in CCR2 + monocyte frequency was also evident in PD patients (B) and correlated with disease duration (C). No difference was observed for CXCR3 + (D) or CD16 + (E) monocytes. (F, G) B‐ and T‐lymphocyte populations do not differ between control and PD samples (n = 50; analysis by Mann‐Whitney test). Data are shown relative to an internal control sample run in each of 8 experiments, then multiplied by the average frequency for the internal control sample to represent actual frequencies. [Color figure can be viewed at http://wileyonlinelibrary.com]

To assess changes in PBMC populations within the Oxford Discovery Cohort, we began by analyzing PBMC subtypes with a panel of cell surface markers (Table S2). Using flow cytometry, we compared the relative frequencies of PBMC subtypes between 50 PD patients and 50 age‐ and sex‐matched controls. Interestingly, a higher frequency of monocytes (identified as CD14+) was found to be present in the PD patients compared with controls (Fig. 1A). To further characterize this monocyte population, we looked at the nonclassical monocyte marker CD16 (FcγIII receptor) and 2 receptors for chemokines known to be elevated in PD: CCR2 (CD192) and CXCR3 (CD183).^21^ A higher frequency of chemotactic CCR2 + monocytes was observed for PD patients compared with controls, whereas no differences in CD16 or CXCR3 monocyte populations were found (Fig. 1B‐E). Furthermore, there was a significant positive correlation between the frequency of CCR2 + monocytes and disease duration within PD patients (*P* = 0.0345) (Fig. 1C). These changes in blood cell frequency were specific for innate immune cells, as no difference in frequency was observed for the CD19 + B‐cell and CD3 + T‐cell lymphocyte subtypes (Fig. 1F,G). Furthermore, the CD8 marker of cytotoxic T cells and the chemokine receptor CXCR3 were found to be similarly expressed on CD3 + T cells, as were markers of senescent (CD57+) and nonsenescent (CD28+) CD8 + T cells (Fig. S3).

### Parkinson's Patient Monocytes Have Increased Mitochondrial Reactive Oxygen Species and Reduced Mitochondrial Content

**Figure 2:**
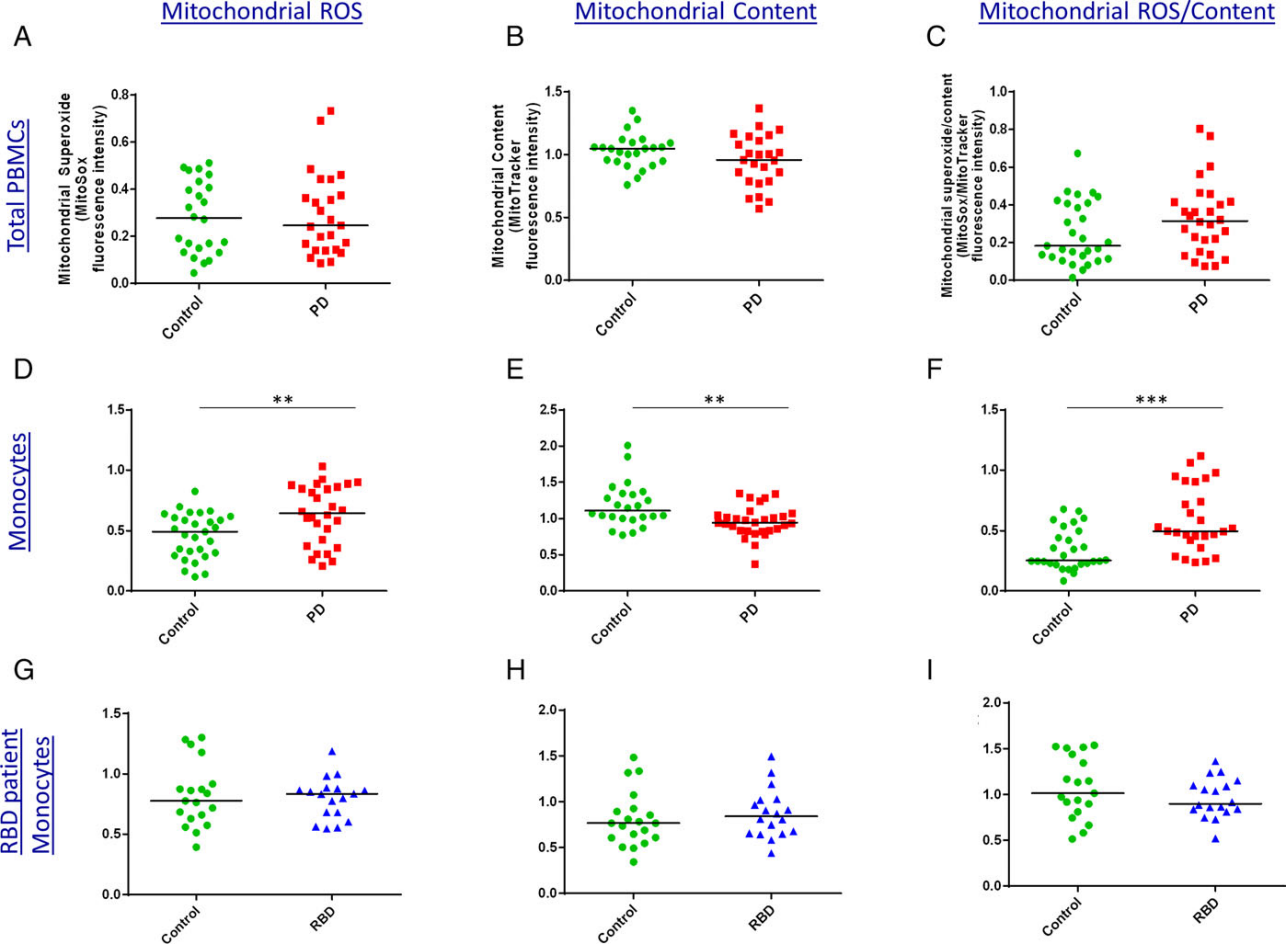
Increased mitochondrial ROS and reduced mitochondrial content in PD patient monocytes. Levels of mitochondrial ROS production (MitoSox) and mitochondrial content (MitoTracker) were assessed in control and PD patient PBMC samples by flow cytometry. (A‐C) Significant differences were not apparent in total PBMCs. (D‐F) However, in the monocyte subset, PD monocytes had greater mitochondrial ROS production and lower mitochondrial content compared with controls. Data are shown relative to rotenone‐treated positive control (n = 25; analysis by Mann‐Whitney test). (G‐I) Mitochondrial ROS and content measured in controls and RBD patients who have a high risk of developing PD (n = 19 controls and 18 RBDs). [Color figure can be viewed at http://wileyonlinelibrary.com]

Many lines of evidence point to mitochondrial dysfunction as a key mediator of neuronal death in PD.^13^ We investigated mitochondrial pathology in control and PD patient peripheral monocytes, as well as lymphocytes and total PBMCs, by measuring mitochondrial content (MitoTracker) and mitochondrial reactive oxygen species (ROS) production (MitoSox). In total PBMCs, mitochondrial content and mitochondrial ROS production were similar across patients and controls (n = 25; Fig. 2A‐C). However, the monocyte subset in PD patients showed striking mitochondrial differences. In PD patient monocytes, there was significantly lower mitochondrial content and higher levels of mitochondrial ROS. Furthermore, when ROS levels were analyzed per mitochondrial content (ie, MitoSox/MitoTracker), the PD patient monocytes had even greater mitochondrial ROS levels (Fig. 2D‐F).

Given this difference in controls versus patients, we examined monocytes from REM‐sleep behavior disorder (RBD) patients, who have a high risk of developing PD. However, in this prodromal group we did not find evidence for reduced mitochondrial content or increased mitochondrial ROS production in monocytes (n = 18; Fig. 2G‐I).

### Reduced Expression and Function of Superoxide Dismutase in PBMCs From Parkinson's and RBD Patients

**Figure 3:**
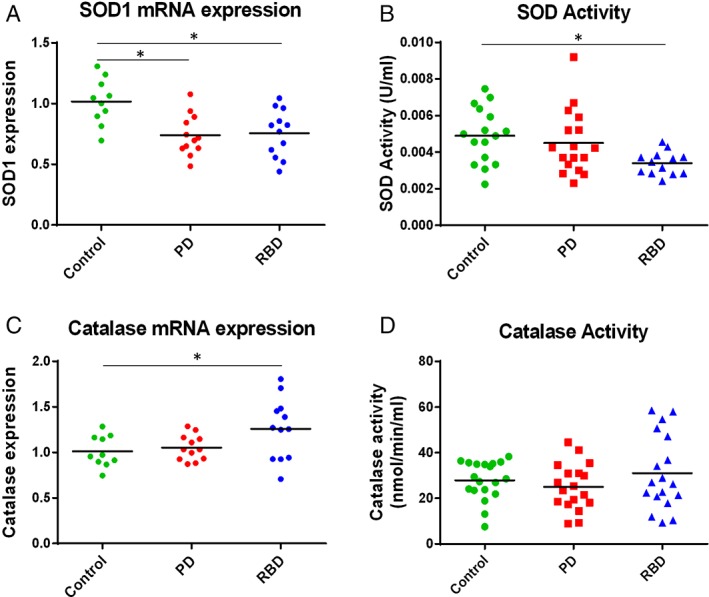
Reduced expression and function of superoxide dismutase (SOD) in disease compared with control PBMCs. (A) SOD1 mRNA expression in PBMCs was quantified using reverse‐transcriptase qPCR. SOD1 levels were significantly lower in PD and RBD patient PBMCs compared with controls (n ≥ 10). (B) Total SOD activity was significantly lower in RBD patient PBMCs compared with controls; analysis by 1‐way ANOVA (n ≥ 13). (C) RBD patient PBMCs had higher catalase mRNA expression compared with controls (n ≥ 10); however no difference was found in catalase activity (D; n = 18). [Color figure can be viewed at http://wileyonlinelibrary.com]

As superoxide is the predominant mitochondrial‐generated ROS,^22^ we further investigated superoxide regulation in PBMCs. Levels of superoxide are in part regulated by the antioxidant enzyme superoxide dismutase (SOD), which catalyzes the conversion of superoxide to oxygen and hydrogen peroxide. To assess whether the increase in mitochondrial ROS in monocytes from PD patients was accompanied by changes in SOD, we looked at the expression of SOD in PBMCs. A reduction in SOD1 transcript levels was observed for both PD and RBD patient PBMCs compared with controls (Fig. 3A). Furthermore, when we assessed the activity of total SOD (SOD 1 and SOD 2) in PBMCs, we found a significant reduction in total SOD activity in individuals with RBD compared with controls (Fig. 3B).

The hydrogen peroxide formed from superoxide by SOD is then converted to oxygen and water by the antioxidant enzyme catalase. We observed increased expression of catalase in PBMCs only in RBD samples compared with controls. No difference in catalase activity was observed between the control and either disease state (Fig. 3C,D), highlighting a deficit in SOD enzymes in patient blood cells.

### Levels of Glycolysis Are Higher in Both Parkinson's and RBD Patient PBMCs

Given the increase in mitochondrial ROS and reduced mitochondrial content observed in PD patient monocytes, we further investigated cellular bioenergetics by measuring oxidative phosphorylation (OCR) and glycolysis (extracellular acidification rate; ECAR) in PBMCs from controls and PD and RBD patients with a Seahorse XF^e^96 Extracellular Flux Analyzer.

**Figure 4:**
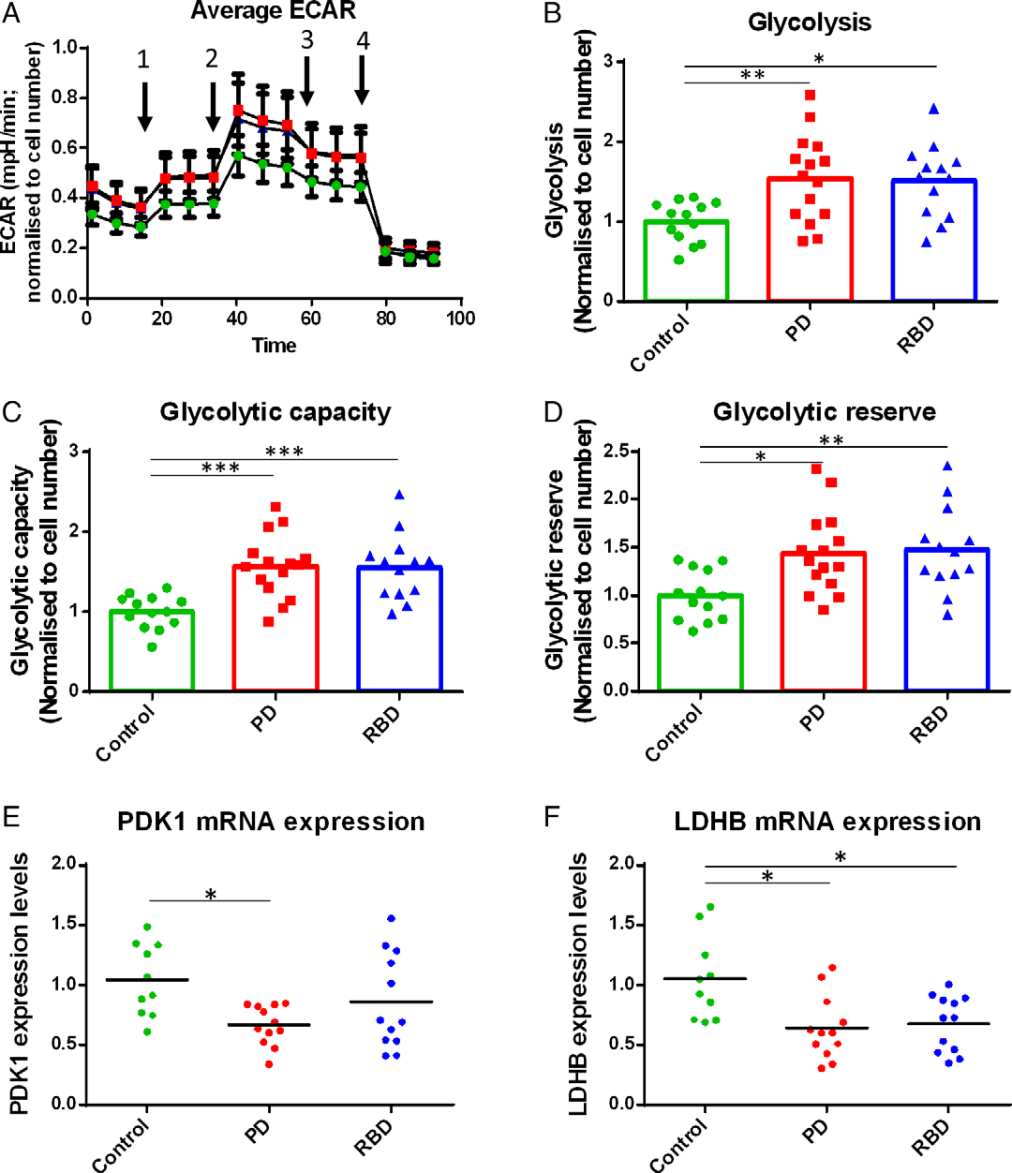
Levels of glycolysis are increased in PD and RBD patient PBMCs compared with controls. (A‐D) Control, PD, and RBD patient PBMCs were analysed using a Seahorse Extracellular Flux Analyzer to measure glycolysis rates. (A) Average traces over time (green, controls; red, PD patients; blue, RBD patients). Arrows indicate sequential injection of oligomycin, FCCP, antimycin A + rotenone, and 2‐deoxyglucose. (B‐D) Measures of glycolysis (extracellular acidification rate) were higher in the PBMCs of PD and RBD patients compared with age‐ and sex‐matched controls. Data are from 3 independent experiments normalized to the mean of the control values for each experiment (14 control, 15 PD, and 13 RBD samples; analysis by 1‐way ANOVA). (E, F) Glycolysis genes pyruvate dehydrogenase kinase 1 (*PDK1*) and lactate dehydrogenase B (*LDHB*) were quantified using reverse‐transcriptase PCR in disease and control PBMCs; analysis by 1‐way ANOVA.

There were no differences in oxygen consumption rates between PBMCs from control and disease groups (Fig. S5). Conversely, we observed a marked elevation in glycolysis for both the PD and RBD patient groups compared with controls, finding glycolysis, glycolytic capacity, and glycolytic reserve all significantly higher in PBMCs from PD and RBD patients compared with controls (Fig. 4A‐D). As monocytes are known to have higher levels of glycolysis than lymphocytes,^23^ we wished to determine whether higher monocyte number may contribute to the increased glycolysis observed in patient PBMCs. Parallel flow‐cytometric analysis did not show a significant difference in monocyte frequency between these control and patient PBMCs sampled for the Seahorse Extracellular Flux Analyzer experiment (n = 15), suggesting that the glycolysis phenotype observed here may be a result of disease‐related cellular changes independent of increased numbers of monocytes.

To further investigate the increased glycolysis observed in the PD and RBD patient PBMCs, we measured transcript levels of enzymes required for glycolysis and found dysregulated expression of 2 glycolysis genes. Pyruvate dehydrogenase kinase 1 (*PDK1*) was significantly reduced in PD patients compared with controls, and lactate dehydrogenase B (*LDHb*) was found to be reduced in both PD and RBD patients (Fig. 4E,F).

We next studied glucose uptake by measuring cell surface expression of GLUT‐1 and the uptake of the fluorescent glucose analogue 2‐NBDG. GLUT1 expression and 2‐NBDG uptake were assessed for total PBMCs, lymphocytes only, and monocytes only. No significant differences were found between disease groups (Fig. S6), indicating that glucose uptake was not driving the increased glycolysis observed for PD and RBD patient PBMCs.

### High‐Throughput Gene Expression Analysis Reveals Reduced COX4 Expression in RBD Patient Monocytes and Lymphocytes

Monocytes are known to become more glycolytic when they become “activated.”^24^ To assess the contribution of monocyte activation to the high rates of glycolysis in PD and RBD patient PBMCs, high‐throughput gene expression analysis of monocytes fluorescence‐activated cell sorted (FACS) from PBMC samples was performed using a Fluidigm Dynamic Array. This transcriptomic analysis did not find evidence for increased monocyte activation in PD or RBD patients compared with controls based on expression of several cytokines and chemokines, cell surface receptors, complement components, and enzymes (Table S4), indicating that the increased glycolysis is independent of increased monocyte activation.

**Figure 5:**
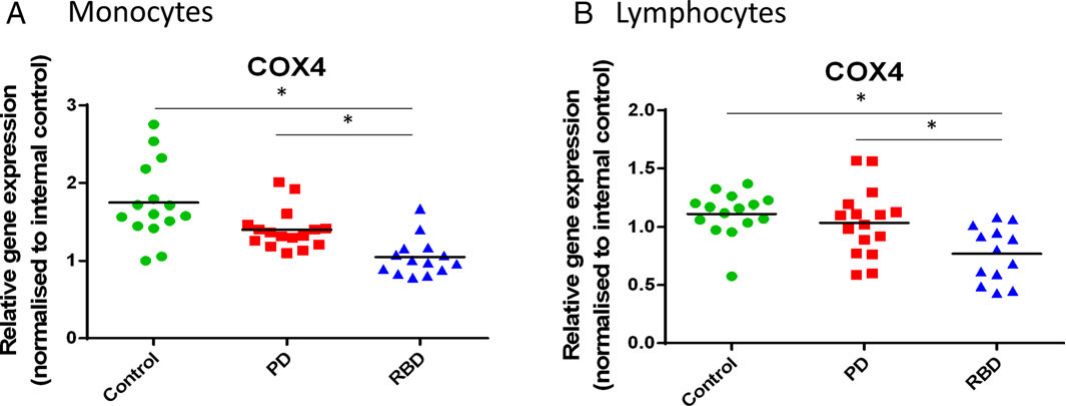
High‐throughput gene expression analysis reveals *COX4* gene expression changes for RBD patients in both monocytes and lymphocytes. PBMCs were sorted for CD14 + monocytes and CD14‐ lymphocytes, and high‐throughput gene expression was performed using a Fluidigm 48.48 Dynamic Array. Cytochrome c oxidase subunit IV isoform 1 (COX4) was significantly reduced in both monocytes (A) and lymphocytes (B) for RBD patients (15 controls, 16 PD patients, and 13 RBD patients). [Color figure can be viewed at http://wileyonlinelibrary.com]

Using this high‐throughput gene expression platform we also analyzed a panel of glycolysis and oxidative stress genes in FACS‐sorted monocytes and lymphocytes. The most striking gene expression difference between disease groups was observed in both monocytes and lymphocytes. The RBD group had significantly lower expression of the mitochondrial enzyme cytochrome c oxidase subunit IV isoform 1 (COX4) compared with both controls and PD patients (Fig. 5), suggesting a unique change in expression in a key gene for mitochondrial function in RBD patients.

## Discussion

This study has revealed differences in key cellular functions in blood cells between PD and RBD patients and healthy controls. Both PD and at‐risk RBD patients exhibited significant increases in PBMC glycolysis and deficits in superoxide dismutase. In the monocyte subset in particular, PD patients exhibited increased mitochondrial ROS and reduced mitochondrial content, in addition to an increased frequency of chemotactic monocytes found in PD patients.

The first aim of this study was to characterize PBMC subpopulations in PD patients compared with control individuals. We found an altered monocyte subpopulation in PD patients with, on average, a higher frequency of total CD14 + monocytes and a higher frequency of monocytes expressing the CCR2 receptor for monocyte chemoattractant protein‐1 (MCP‐1 or CCL2). In addition, the CCR2 + monocyte population in PD patients significantly increased with disease duration. Together with other studies,^21^, ^25^ this work shows that the CCR2 chemokine receptor is specifically dysregulated in monocytes in PD and is likely to play a role in the immune component of PD. Monocytes expressing the CCR2 chemokine receptor may be recruited into PD brain tissue, as brain cells have been shown to produce the CCR2 ligand MCP‐1.^26^, ^27^ If they are proinflammatory, these monocytes may be detrimental to brain health; however, more studies are required to investigate the role of peripheral monocyte recruitment into the human brain throughout disease progression. Increased monocyte frequency may indicate dominance of the innate immune system, which has been shown to occur with aging.^28^, ^29^ Although a recent study by Cook et al (2017) did not find a significant increase in CD14 + monocyte frequency in PD patients (albeit with a smaller sample size), there was a trend toward an increase. In addition, they observed a decrease in T‐cell frequency, corroborating the idea of dominance of the innate immune system. In addition, levels of the PD‐related protein LRRK2 were found to correlate with PD patient monocytes.^8^ Monocytes are certainly emerging as a key cell type conferring neurodegenerative disease risk, and several monocyte‐specific genetic disease variants are being identified.^30^, ^31^

In addition to increased monocyte frequency, this study also demonstrates monocyte‐specific mitochondrial dysfunction in PD patients. Mitochondrial dysfunction has been demonstrated in many PD models including human neurons and fibroblasts.^13^, ^32^, ^33^, ^34^ We investigated whether mitochondrial dysfunction and oxidative stress were also detectable in easily accessible PBMCs from PD patients and, importantly, RBD patients. We found a substantial increase in mitochondrial ROS in monocytes of PD patients. The accompanying reduction in mitochondrial content suggests that there may be fewer functioning mitochondria and may indicate a reduced capacity to replace dysfunctional mitochondria. These perturbations suggest mitochondrial dysfunction may contribute to pathology.

These results expand on previous literature indicating generally increased oxidative stress in the peripheral blood of PD patients. In neutrophil granulocytes, PD patients were found to have altered mitochondrial mass and membrane potential, as well as increased oxidative stress.^35^ Prigione et al (2006) found increased total ROS levels in PD patient PBMCs compared with controls,^36^ and a recent pilot study demonstrated lower whole‐blood levels of reduced glutathione in RBD as well as PD patients.^37^

Given the increased mitochondrial oxidative stress observed in the PD patient PBMCs, we further investigated mitochondrial function. We found that although PD and RBD patient PBMCs had similar oxygen consumption rates, they had significantly increased glycolysis compared with controls. All the Seahorse Extracellular Flux Analyzer experiments in this study were performed in the presence of oligomycin and rotenone/antimycin A to inhibit oxidative phosphorylation, resulting in a measurement specific to glycolysis.^38^

Interestingly, the RBD patients resembled the PD patients more closely than the controls, demonstrating the predictive value of these findings and the future possibility of establishing a glycolysis‐related biomarker for the early stages of PD. PET imaging with ^18^F‐fluorodeoxyglucose also showed changes in brain glucose metabolism in RBD patients compared with controls, with most brain areas showing increased metabolism.^39^, ^40^ Perturbed energy production may be upstream of many other functional cellular defects; however, further studies are required to establish a causal chain of events.

Furthermore, we have shown that the glycolysis disease phenotype is not driven by monocyte activation or glucose uptake. Reduced COX4 expression may contribute to defective mitochondrial function and a propensity toward glycolysis as a preferred energy production pathway. COX4 was not significantly reduced in the PD patient group, suggesting that COX4 may be part of an early pathological mechanism in these cells. Peripheral molecular changes specific to the early stages of disease have also been observed for other disorders.^41^, ^42^ Nevertheless, this result awaits validation in a larger cohort of both PD and RBD patients.

Recently, glucose regulation and energy metabolism have emerged as major players in neurological disease and specifically in dopaminergic disorders. Glycolysis pathway disruption has been identified in PBMCs from schizophrenic patients^43^, ^44^ and analysis of serum metabolites in patients with restless legs syndrome (RLS) showed several dysregulated pathways, including increased lactate in both RLS and PD.^45^ Vascular degeneration has also been observed in PD brain tissue.^46^ A study complementary to our own was recently published by Maynard et al (2015) on Alzheimer's patient PBMCs. Although no differences in glycolytic reserve or MitoSox were found, reduced proton leak and DNA repair activity in AD patients were reported.^47^ Future studies investigating multiple patient groups will aid in identifying common and diverging PBMC pathology across neurodegenerative diseases. Our study is among the first to present peripheral blood cell phenotypes of RBD patients alongside PD patients, providing a unique opportunity to gather information about early disease processes in humans. The significant changes in SOD and glycolysis in RBD patients, prior to clinical signs of PD, demonstrate the predictive value of including a prodromal group in studies of early disease pathology. It is interesting to note that RBD patients can have greater phenotypes than PD patients on some measures, most striking here in SOD activity and COX4 expression. This is in keeping with an emerging idea that the presence of RBD preceding PD is potentially a more severe phenotype than PD in the absence of RBD.^48^, ^49^ It is also important to note that in our cohort PD patients were medicated with l‐dopa at a range of doses (Table S1). We investigated the relationship between l‐dopa dose with the PBMC variables measured and found no effect (Fig. S7). Drug‐naive patients also did not cluster separately from other patients. However, we cannot rule out that other unreported medication could have an effect on the processes measured.

Despite the novel findings presented here, the restricted volume of blood drawn from each participant resulted in limited numbers of patient cells, preventing all assays from being carried out with the same samples. For this reason, it was not feasible to conduct protein analysis or measure glycolysis rates using sorted PBMC cell populations, which would provide further information about the bioenergetics of specific cell types and their relative contributions, which is masked in analyses of total PBMCs.^23^ For each experiment we used the largest sample size possible (determined by availability of patient samples) while maintaining age‐ and sex‐matched groups. Further studies are required to fully understand the relationship between functional and gene expression changes in glycolysis and inflammation in specific cell types of PBMCs in the context of PD. This will be key to understanding the potential use of PBMCs as biomarkers and therapeutic targets. Nevertheless, despite modest sample sizes, this work demonstrates significant changes in PBMC mitochondrial function and energy production.

In conclusion, this study presents clear mitochondrial and glycolysis changes in prodromal and early PD patient blood cells. This work demonstrates PBMCs as an easily accessible cell type with which to quantitatively measure early physiological changes by critically working with human tissue.

## Authors’ contributions

A.M.S. and R.W.M. conceived and designed the experiments and interpreted data. A.M.S. performed PBMC isolation and cellular and molecular experiments and wrote the article. C.D. and A.M.S. performed Seahorse and qPCR experiments. B.J.R. performed Seahorse experiments, advised on experimental design, and revised the article. G.I.J advised on experimental design, interpreted data, and revised the article. J.A.A. revised the article. S.E. coordinated collection of blood samples. M.R., F.B., and C.R. collected blood samples and performed clinical examinations. A.K.S. and M.H. advised on experimental design, interpreted data, and revised the article. All the authors have read and approved the final article.

## Supporting information

Additional Supporting Information may be found in the online version of this article at the publisher's web‐site

**SUPPLEMENTARY TABLE 1.** Demographic information of participants

**SUPPLEMENTARY TABLE 2.** Antibodies used for PBMC phenotyping.
PBMC samples were labeled and analyzed with 2 panels of antibodies. Panel 1 consisted of antibodies to CD3, CD8a, CD14, CD16, and CD19 and nonsenescent marker CD28 and senescent marker CD57. Panel 2 consisted of antibodies to CD3, CD8a, CD14, CD16, CD19, and chemokine receptors CD183 (CXCR3) and CD192 (CCR2).

**SUPPLEMENTARY TABLE 3.** qRT‐PCR primers

**SUPPLEMENTARY TABLE 4.** TaqMan gene expression assays for Fluidigm Dynamic Array high‐throughput qPCR

**SUPPLEMENTARY FIG. 1.** Outline of principal experiments. Fifteen milliliters of blood was collected from each participant, and PBMCs were isolated for experiments. n, number of individual patient and control samples used for each experiment. Because of limited numbers of samples, later experiments were performed with a reduced number of matched patient and control samples. Patient samples used in experiments were selected purely on and age‐ and sex‐matching criteria for the control samples available.

**SUPPLEMENTARY FIG. 2.** Gating for flow‐cytometry experiments. (a) Gating of total PBMCs, monocytes, and lymphocytes to exclude cell debris. (b) Gating of single cells to exclude doublets. (c) Gating of live cells by excluding dead cells that are positive for Live/Dead dye. (d) Gating of T cells using the cell surface marker CD3. The positive population is easily distinguished from the other cells (B cells and monocytes) in the PBMC sample. (e) MitoSox staining separation from unstained control.

**SUPPLEMENTARY FIG. 3.** No differences in T‐lymphocyte subsets were found in control and PD patient PBMCs. Investigation of T‐cell subsets showed no significant difference between control and PD samples in frequency of (a) CD8 + or (b) CD8+CXCR3 + T cells. No difference in the frequency of (c) senescent (CD57+) or (d) nonsenescent (CD28+) CD8 + T cells was found between controls and PD patients.

**SUPPLEMENTARY FIG. 4.** No difference in mitochondrial membrane potential in control individuals and PD patients. Mitochondrial membrane potential measured in (a) PBMCs and (b) monocytes in controls and PD patients by TMRM.

**SUPPLEMENTARY FIG. 5.** Oxygen consumption rates are similar in control, PD, and RBD patient PBMCs. Control, PD, and RBD patient PBMCs were analyzed using a Seahorse Extracellular Flux Analyzer to measure oxygen consumption rates. (a) Average traces over time (green, controls; red, PD patients; blue, RBD patients). Arrows indicate sequential injection of oligomycin, FCCP, and antimycin A + rotenone. (b) Plot of OCR versus ECAR data for controls, PD patients, and RBD patients. No significant difference was found in the measures of (c) basal respiration, (d) maximal respiration, (e) spare capacity, (f) ATP production, or (g) nonmitochondrial respiration. Data are from 3 independent experiments, normalized to the mean of the control values for each experiment (14 control, 15 PD, and 13 RBD samples).

**SUPPLEMENTARY FIG. 6.** Expression of GLUT1 glucose transporter and uptake of fluorescent glucose analogue 2‐NBDG was similar in control, PD, and RBD patient PBMCs. (a‐c) GLUT1 cell surface expression in PBMCs from controls, PD, and RBD patients was measured by flow cytometry and analyzed for (a) total PBMCs or (b) lymphocytes only or (c) monocytes only (18 controls, 18 PD patients, 13 RBD patients; graphs display median). (d‐f) Glucose uptake was assessed by flow cytometry using the fluorescent glucose analogue 2‐NBDG in control and PD and RBD patient samples. Thirty‐minute accumulation of 2‐NBDG for (d) total PBMCs, (e) lymphocytes, (f) monocytes (17 controls, 17 PD patients, 13 RBD patients; graphs display mean).

**SUPPLEMENTARY FIG. 7.** No effect of l‐dopa dosage on monocyte frequency, mitochondrial ROS production, or glycolysis in PD patients. No correlation was found between the l‐dopa‐equivalent dose daily (LEDD) for PD patients and their (a) frequency of CD14 + monocytes, (b) monocytic mitochondrial ROS production, or (c) glycolytic rate of PBMCs.
