# Supplementary figures and images for: Mitochondrial dysfunction and increased glycolysis in prodromal and early Parkinson's blood cells
Source: Mov Disord. 2018 Oct 7;33(10):1580–90. doi: 10.1002/mds.104 (PMC6221131; doi:10.1002/mds.104)

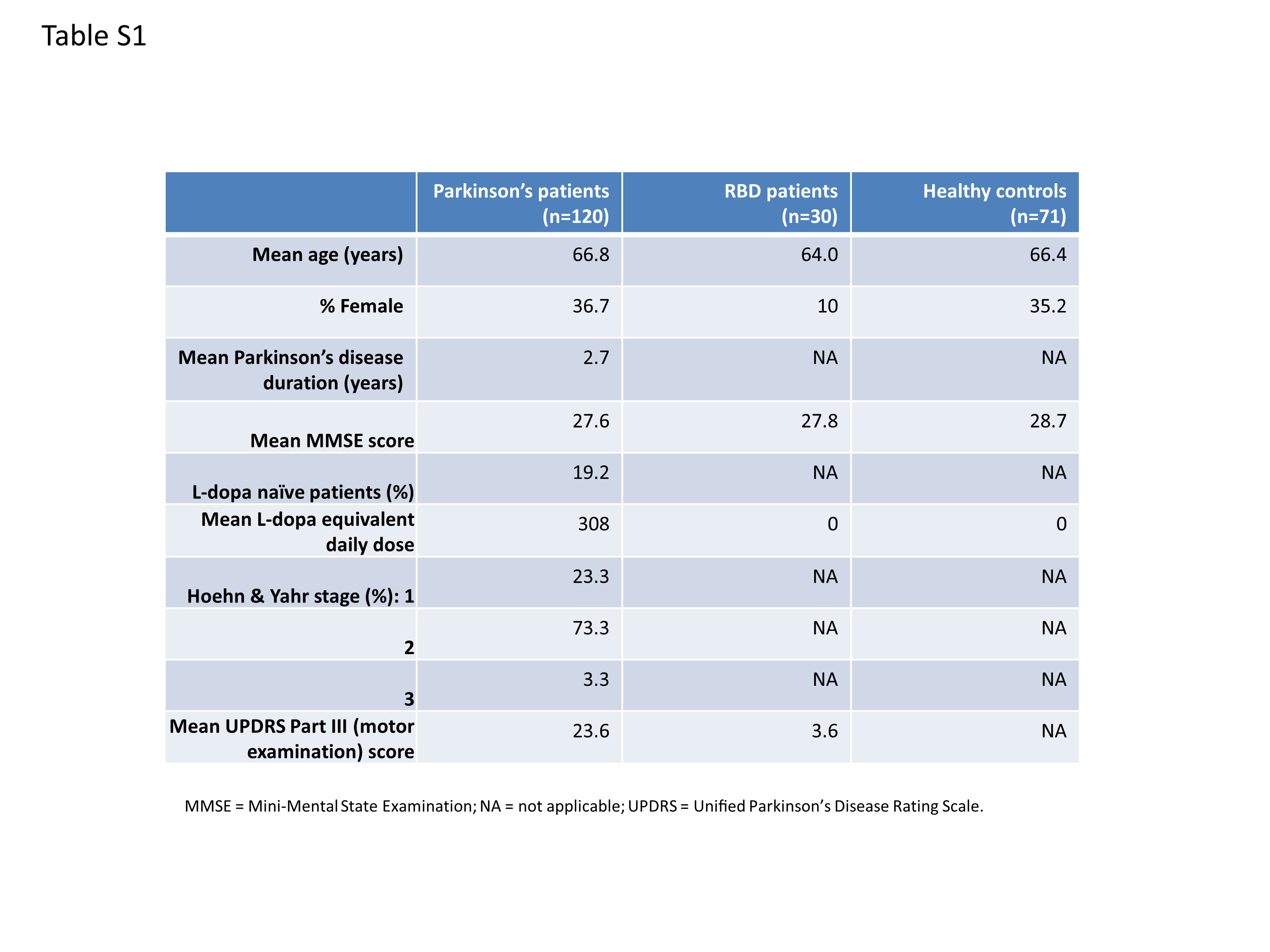

Supplement: Supplementary file 1 — SUPPLEMENTARY TABLE 1. Demographic information of participants [file MDS-33-1580-s001.tif]

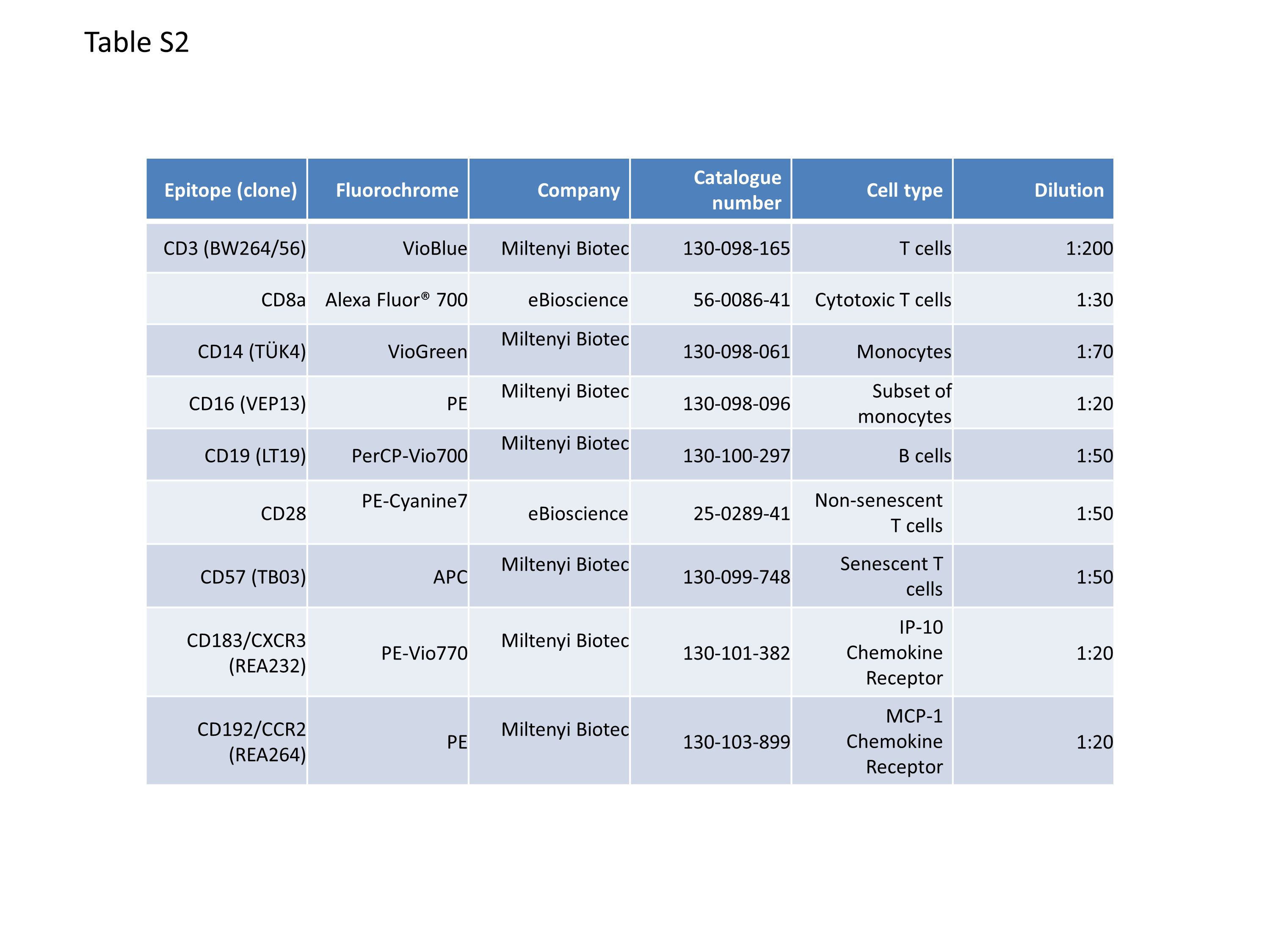

Supplement: Supplementary file 2 — SUPPLEMENTARY TABLE 2. Antibodies used for PBMC phenotyping. PBMC samples were labeled and analyzed with 2 panels of antibodies. Panel 1 consisted of antibodies to CD3, CD8a, CD14, CD16, and CD19 and nonsenescent marker CD28 and senescent marker CD57. Panel 2 consisted of antibodies to CD3, CD8a, CD14, CD16, CD19, and chemokine receptors CD183 (CXCR3) and CD192 (CCR2). [file MDS-33-1580-s002.tif]

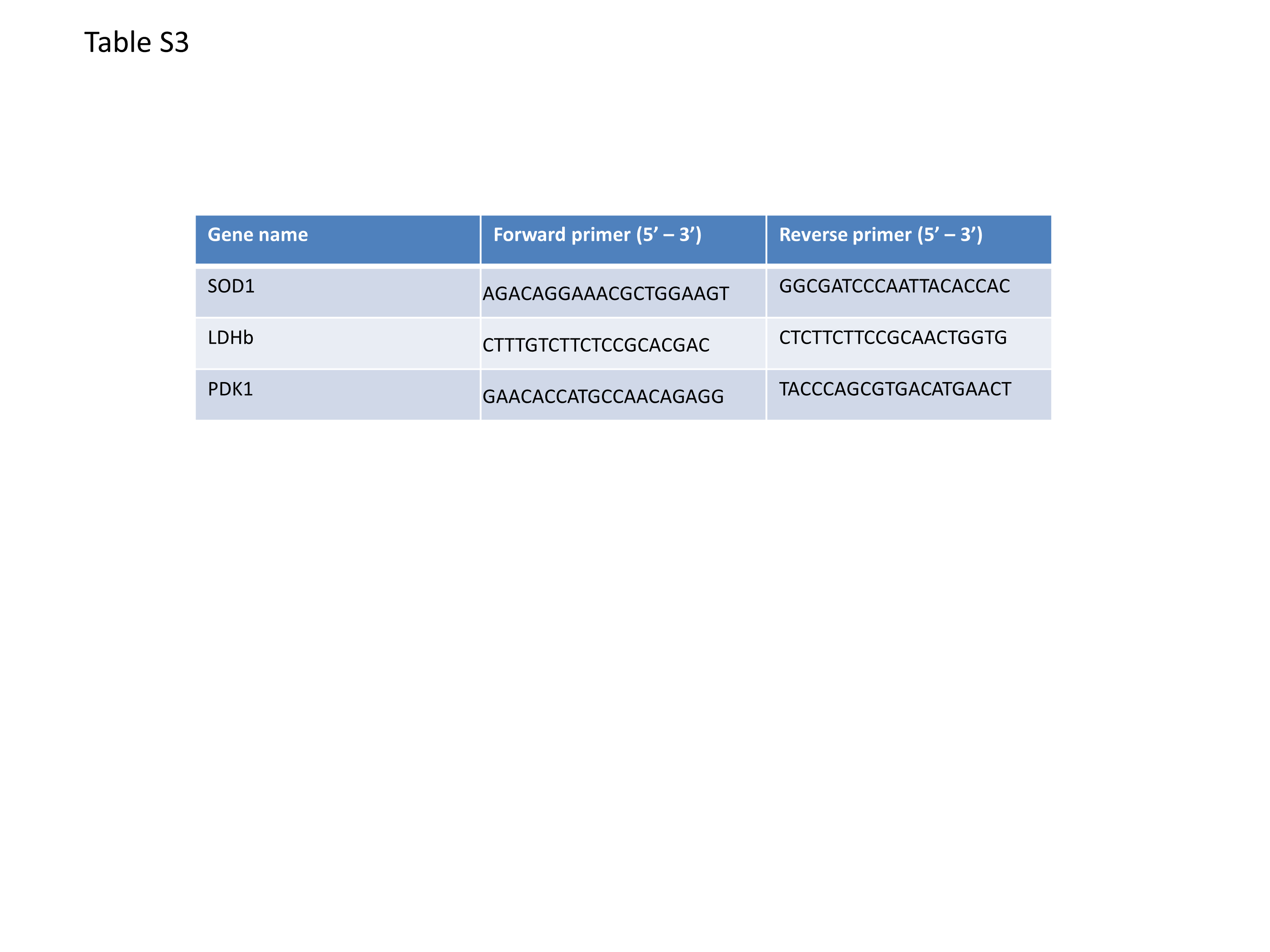

Supplement: Supplementary file 3 — SUPPLEMENTARY TABLE 3. qRT‐PCR primers [file MDS-33-1580-s003.tif]

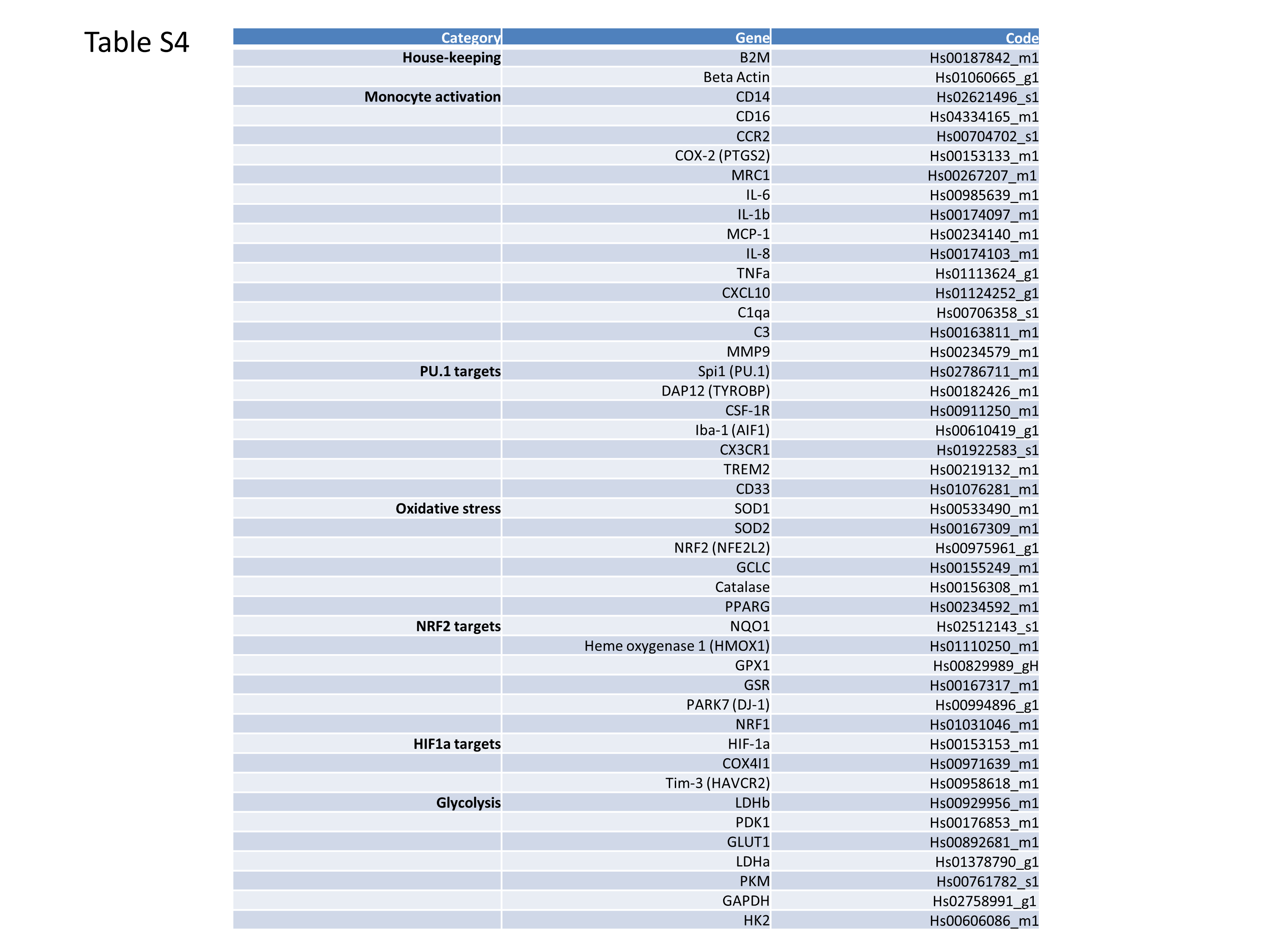

Supplement: Supplementary file 4 — SUPPLEMENTARY TABLE 4. TaqMan gene expression assays for Fluidigm Dynamic Array high‐throughput qPCR [file MDS-33-1580-s004.tif]

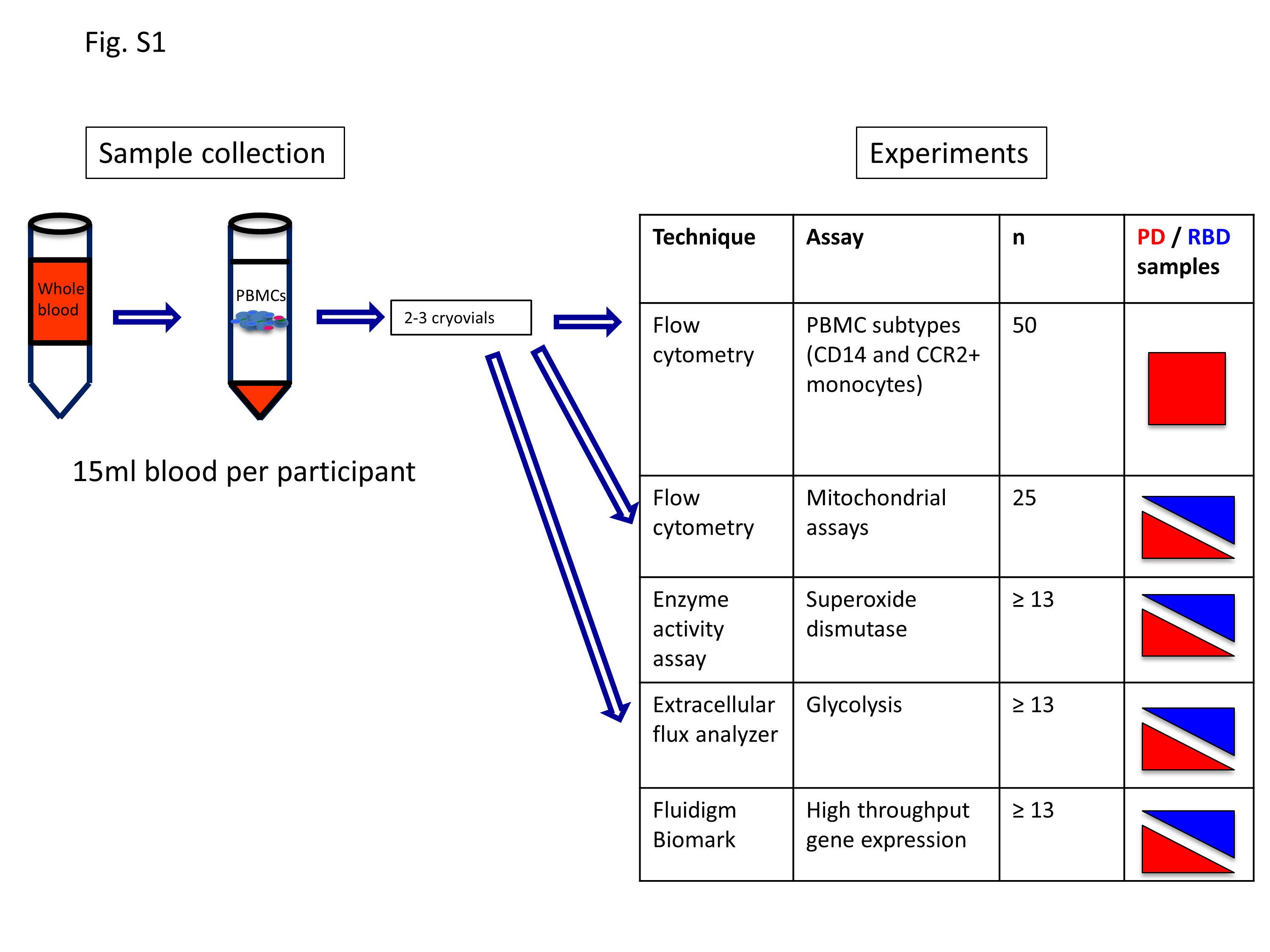

Supplement: Supplementary file 5 — SUPPLEMENTARY FIG. 1. Outline of principal experiments. Fifteen milliliters of blood was collected from each participant, and PBMCs were isolated for experiments. n, number of individual patient and control samples used for each experiment. Because of limited numbers of samples, later experiments were performed with a reduced number of matched patient and control samples. Patient samples used in experiments were selected purely on and age‐ and sex‐matching criteria for the control samples available. [file MDS-33-1580-s005.tif]

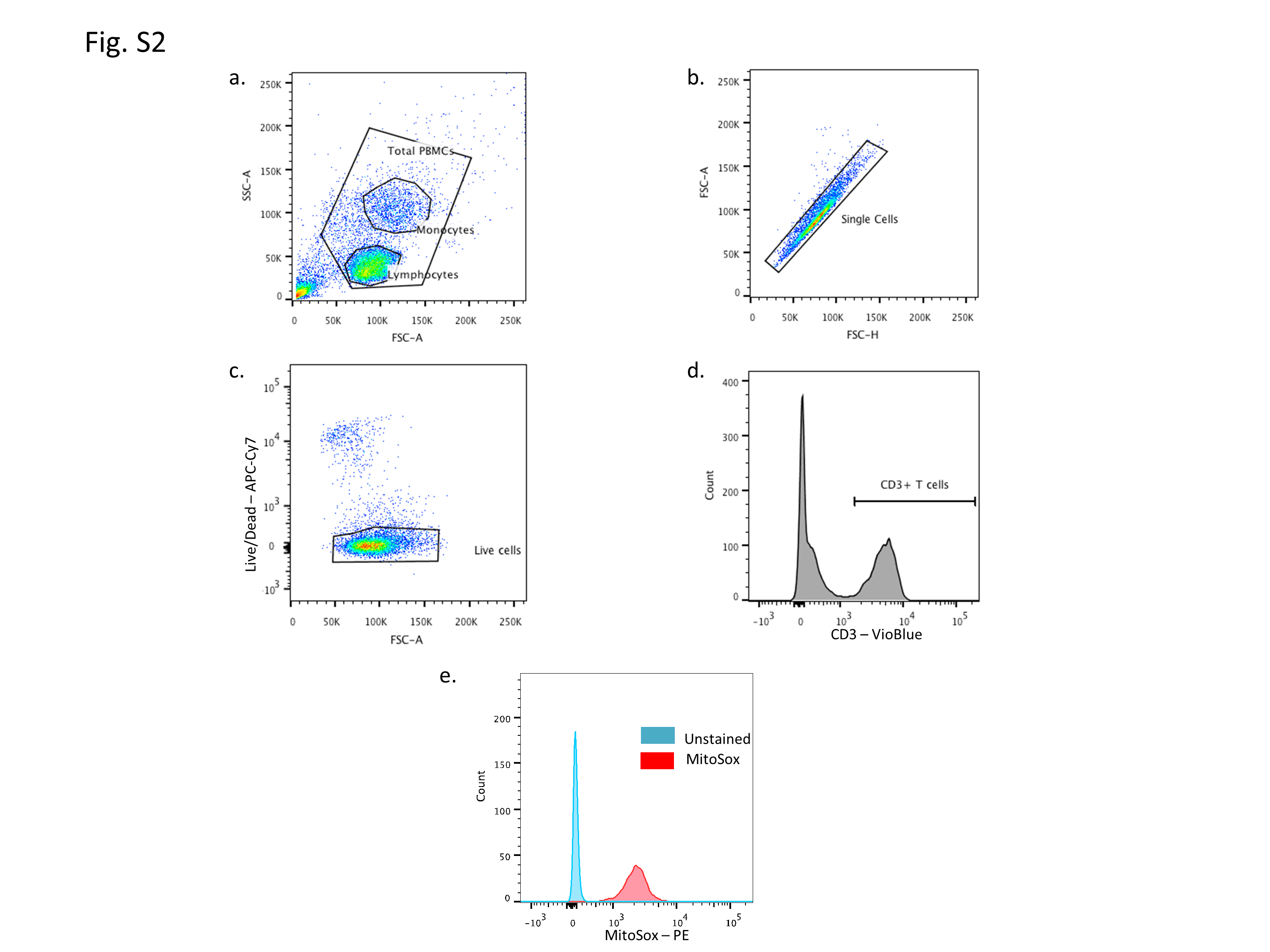

Supplement: Supplementary file 6 — SUPPLEMENTARY FIG. 2. Gating for flow‐cytometry experiments. (a) Gating of total PBMCs, monocytes, and lymphocytes to exclude cell debris. (b) Gating of single cells to exclude doublets. (c) Gating of live cells by excluding dead cells that are positive for Live/Dead dye. (d) Gating of T cells using the cell surface marker CD3. The positive population is easily distinguished from the other cells (B cells and monocytes) in the PBMC sample. (e) MitoSox staining separation from unstained control. [file MDS-33-1580-s006.tif]

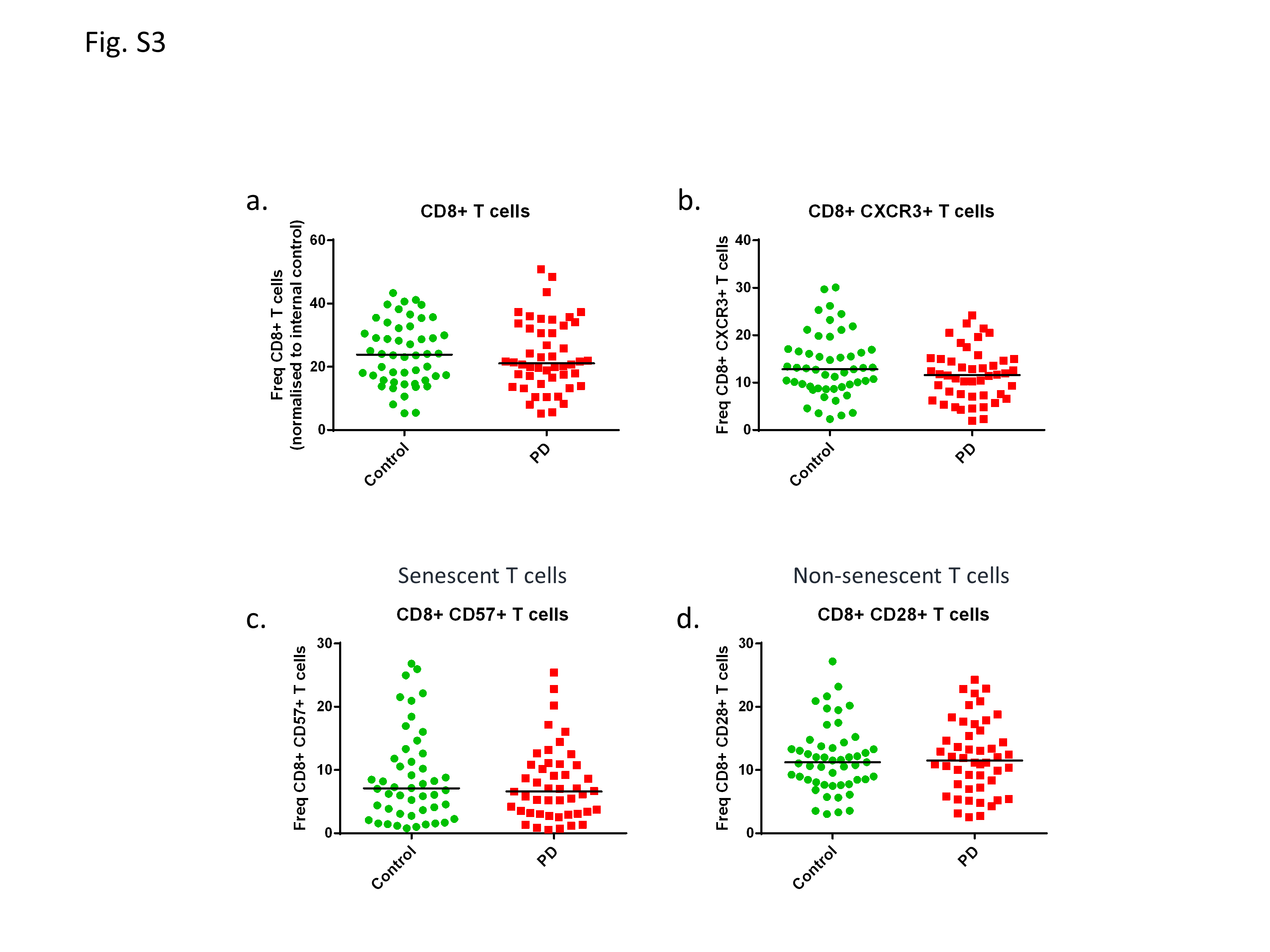

Supplement: Supplementary file 7 — SUPPLEMENTARY FIG. 3. No differences in T‐lymphocyte subsets were found in control and PD patient PBMCs. Investigation of T‐cell subsets showed no significant difference between control and PD samples in frequency of (a) CD8 + or (b) CD8+CXCR3 + T cells. No difference in the frequency of (c) senescent (CD57+) or (d) nonsenescent (CD28+) CD8 + T cells was found between controls and PD patients. [file MDS-33-1580-s007.tif]

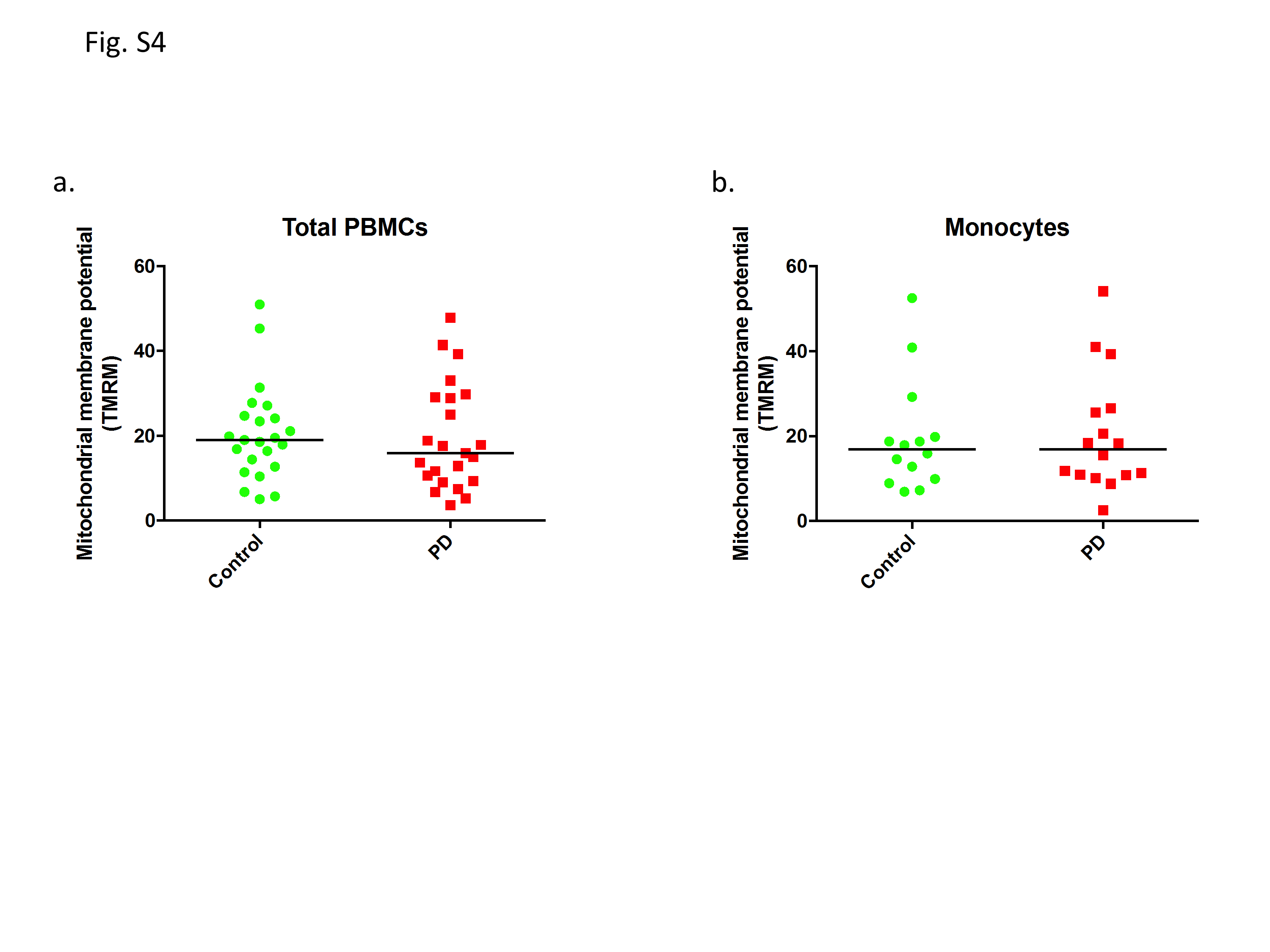

Supplement: Supplementary file 8 — SUPPLEMENTARY FIG. 4. No difference in mitochondrial membrane potential in control individuals and PD patients. Mitochondrial membrane potential measured in (a) PBMCs and (b) monocytes in controls and PD patients by TMRM. [file MDS-33-1580-s008.tif]

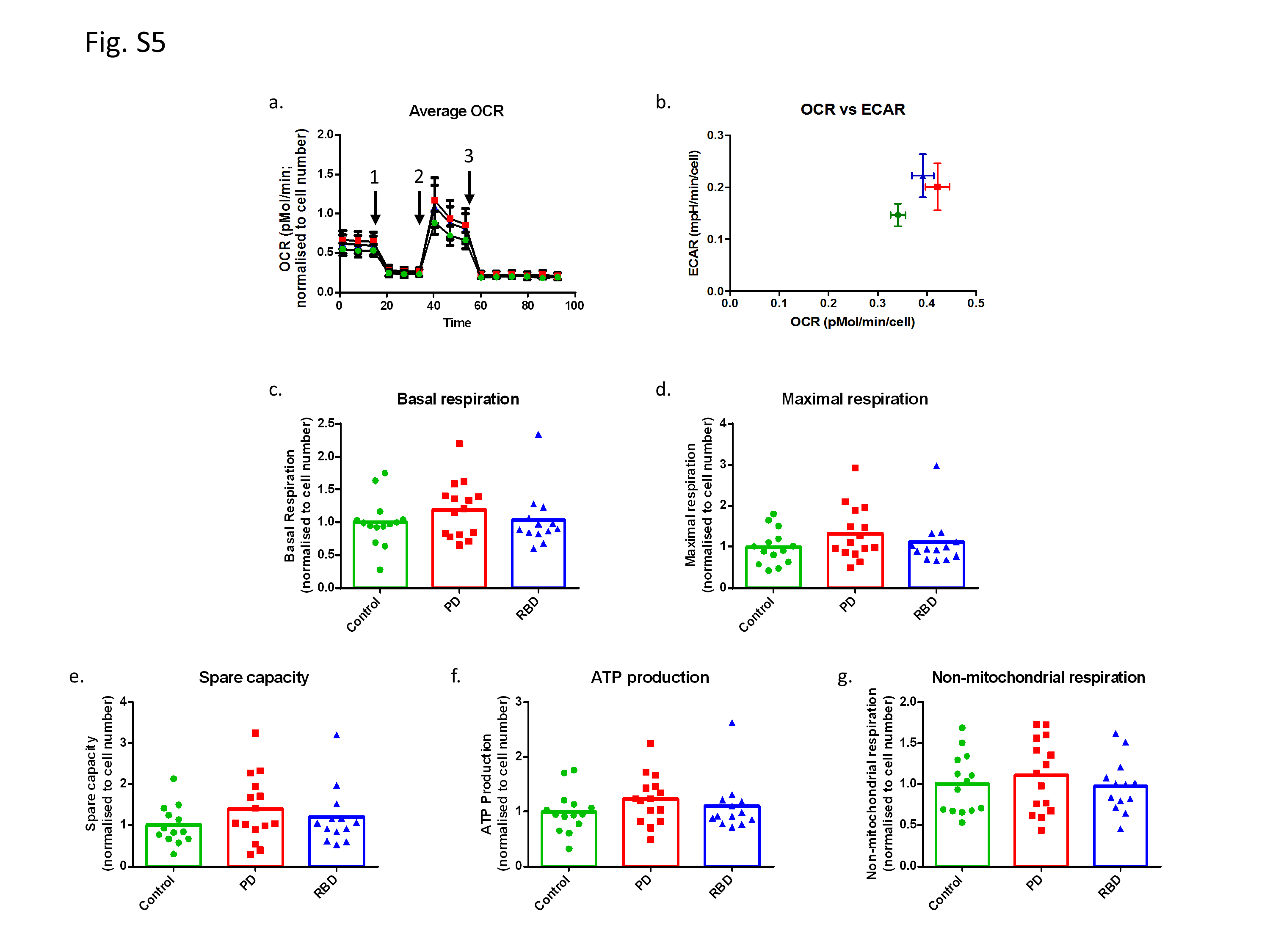

Supplement: Supplementary file 9 — SUPPLEMENTARY FIG. 5. Oxygen consumption rates are similar in control, PD, and RBD patient PBMCs. Control, PD, and RBD patient PBMCs were analyzed using a Seahorse Extracellular Flux Analyzer to measure oxygen consumption rates. (a) Average traces over time (green, controls; red, PD patients; blue, RBD patients). Arrows indicate sequential injection of oligomycin, FCCP, and antimycin A + rotenone. (b) Plot of OCR versus ECAR data for controls, PD patients, and RBD patients. No significant difference was found in the measures of (c) basal respiration, (d) maximal respiration, (e) spare capacity, (f) ATP production, or (g) nonmitochondrial respiration. Data are from 3 independent experiments, normalized to the mean of the control values for each experiment (14 control, 15 PD, and 13 RBD samples). [file MDS-33-1580-s009.tif]

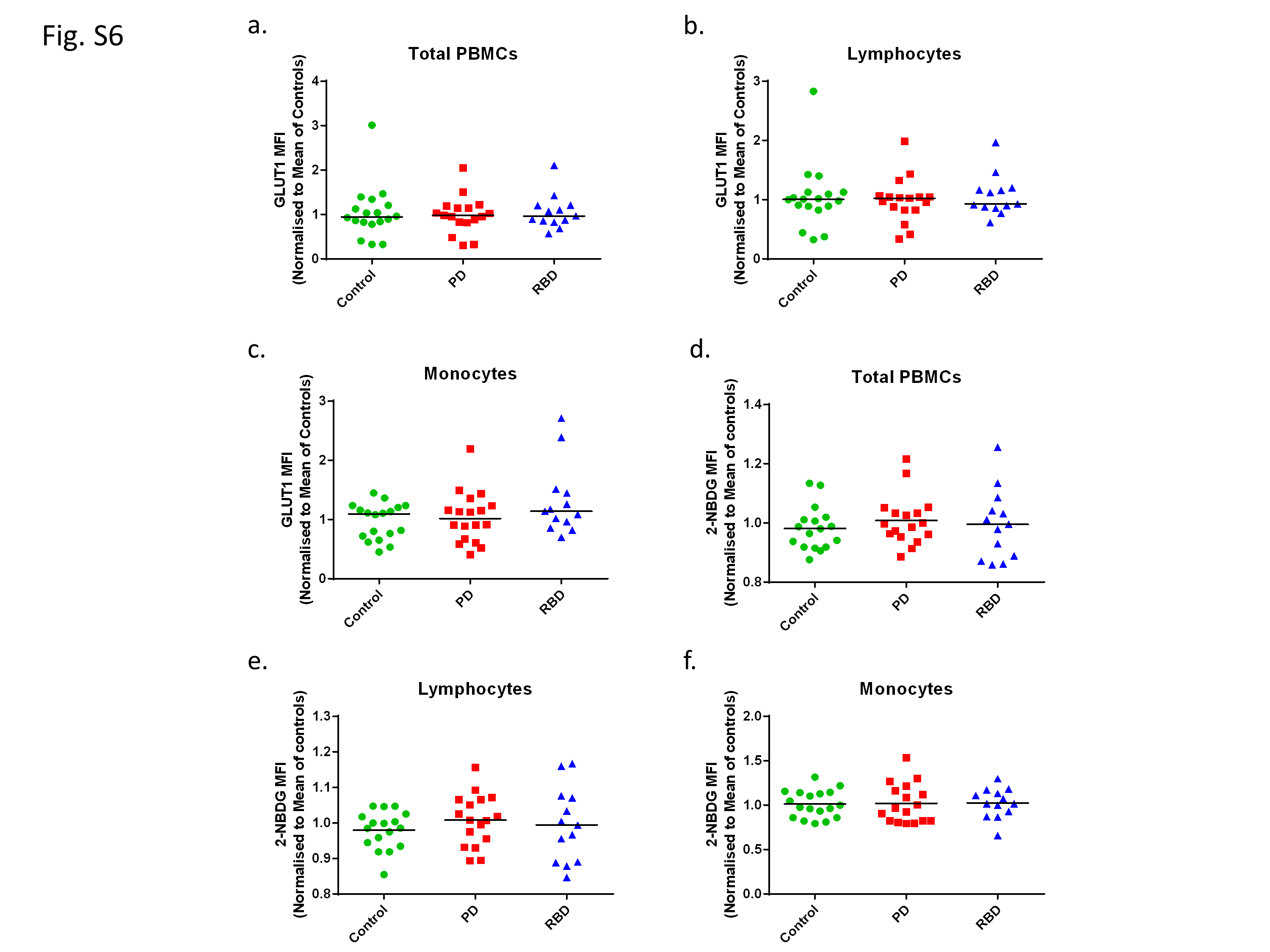

Supplement: Supplementary file 10 — SUPPLEMENTARY FIG. 6. Expression of GLUT1 glucose transporter and uptake of fluorescent glucose analogue 2‐NBDG was similar in control, PD, and RBD patient PBMCs. (a‐c) GLUT1 cell surface expression in PBMCs from controls, PD, and RBD patients was measured by flow cytometry and analyzed for (a) total PBMCs or (b) lymphocytes only or (c) monocytes only (18 controls, 18 PD patients, 13 RBD patients; graphs display median). (d‐f) Glucose uptake was assessed by flow cytometry using the fluorescent glucose analogue 2‐NBDG in control and PD and RBD patient samples. Thirty‐minute accumulation of 2‐NBDG for (d) total PBMCs, (e) lymphocytes, (f) monocytes (17 controls, 17 PD patients, 13 RBD patients; graphs display mean). [file MDS-33-1580-s010.tif]

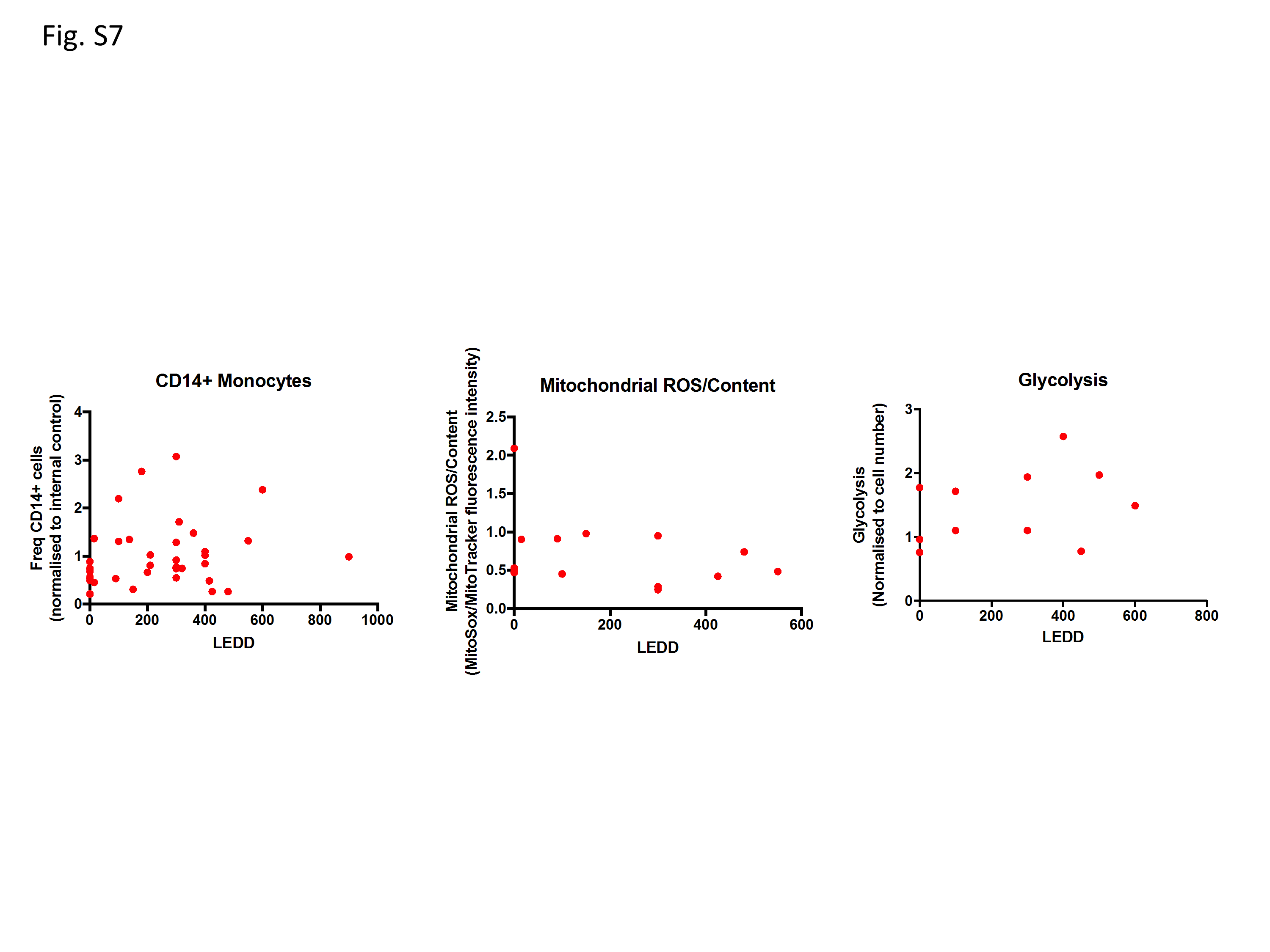

Supplement: Supplementary file 11 — SUPPLEMENTARY FIG. 7. No effect of l‐dopa dosage on monocyte frequency, mitochondrial ROS production, or glycolysis in PD patients. No correlation was found between the l‐dopa‐equivalent dose daily (LEDD) for PD patients and their (a) frequency of CD14 + monocytes, (b) monocytic mitochondrial ROS production, or (c) glycolytic rate of PBMCs. [file MDS-33-1580-s011.tif]
